# Differential serotonergic modulation of two types of aggression in weakly electric fish

**DOI:** 10.3389/fnbeh.2012.00077

**Published:** 2012-11-19

**Authors:** Lucía Zubizarreta, Rossana Perrone, Philip K. Stoddard, Gustavo Costa, Ana C. Silva

**Affiliations:** ^1^Unidad Bases Neurales de la Conducta, Instituto de Investigaciones Biológicas Clemente EstableMontevideo, Uruguay; ^2^Department of Biological Sciences, Florida International UniversityMiami, FL, USA; ^3^Departamento de Neuroquímica, Instituto de Investigaciones Biológicas Clemente EstableMontevideo, Uruguay; ^4^Laboratorio de Neurociencias, Facultad de Ciencias, Universidad de la RepúblicaMontevideo, Uruguay

**Keywords:** agonistic behavior, 5-HT, *Gymnotus omarorum*, *Brachyhypopomus gauderio*, territorial aggression, reproduction-related aggression

## Abstract

Agonistic aggression has provided an excellent framework to study how conserved circuits and neurochemical mediators control species-specific and context-dependent behavior. The principal inhibitory control upon aggression is serotonin (5-HT) dependent, and the activation of 5-HT_1A_ receptors is involved in its action. To address whether the serotonergic system differentially regulates different types of aggression, we used two species of weakly electric fish: the solitary *Gymnotus omarorum* and the gregarious *Brachyhypopomus gauderio*, which display distinctive types of aggression as part of each species' natural behavioral repertoire. We found that in the reproduction-related aggression displayed by *B. gauderio* after conflict resolution, the serotonergic activity follows the classic pattern in which subordinates exhibit higher 5-HT levels than controls. After the territorial aggression displayed by *G. omarorum*, however, both dominants and subordinates show lower 5-HT levels than controls, indicating a different response of the serotonergic system. Further, we found interspecific differences in basal serotonin turnover and in the dynamic profile of the changes in 5-HT levels from pre-contest to post-contest. Finally, we found the expected reduction of aggression and outcome shift in the territorial aggression of *G. omarorum* after 8-OH-DPAT (5-HT_1A_ receptor agonist) administration, but no effect in the reproduction-related aggression of *B. gauderio*. Our results demonstrate the differential participation of the serotonergic system in the modulation of two types of aggression that we speculate may be a general strategy of the neuroendocrine control of aggression across vertebrates.

## Introduction

Animal conflicts arise over limited resources, such as territories, food, and mates (King, 1973). Aggression, defined as an overt behavior which leads to displacing, dominating, or harming another individual, occurs during the contest phase of agonistic interactions (Nelson, 2006). Several attempts to provide a comprehensive classification of aggression include particular types that can be observed in agonistic context: reproduction-related aggression, rank-related aggression, and territorial aggression (Moyer, 1968; Brain, 1979; Wingfield et al., 2006). We now understand that the circuits underlying social behavior may show a specific spatio-temporal pattern of activation according to the type of behavior displayed (e.g., male aggressive behavior vs. male reproductive behavior, Newman, 1999). As a particular case of this prediction, we postulate that each type of aggression relies on a distinct spatio-temporal pattern of activation of this neural network.

Serotonin (5-HT) exerts the principal inhibitory control upon aggression (Nelson and Chiavegatto, 2001). In several classes of vertebrates experimental increases of 5-HT inhibit aggression (Fachinelli et al., 1989; Deckel and Jevitts, 1997; Ferris et al., 1997; Larson and Summers, 2001; Winberg et al., 2001; Perreault et al., 2003; Reist et al., 2003; Sperry et al., 2003). In addition, the 5-HT deficiency hypothesis was formulated based on the correlation between low levels of the 5-HT metabolite 5-hydroxyindoleacetic acid (5-HIAA) and aggressive or violent behavior in humans and other primates (Krakowski, 2003). In several species in which the serotonergic system has been analyzed during agonistic interactions, serotonergic activity increases consistently in subordinates (Yodyingyuad et al., 1985; Blanchard et al., 1991; Summers and Greenberg, 1995; Overli et al., 1999; Summers et al., 2005b) and decreases or remains unchanged in dominants (Van Erp and Miczek, 2000; Ferrari et al., 2003; Summers et al., 2003). Based on these observations, it is generally accepted that high levels of 5-HT inhibit aggression, though the assumption of a simple relationship between 5-HT and agonistic behavior has been challenged (De Boer and Koolhaas, 2005; Caramaschi et al., 2008). The activation of 5-HT_1A_ receptors has been involved in the inhibitory effect of 5-HT on aggression (Nelson and Chiavegatto, 2001; Nelson and Trainor, 2007). Administration of agonists of the 5-HT_1A_ receptor inhibits agonistic behavior and/or induces submissive displays in teleosts (Clotfelter et al., 2007; Allee et al., 2008), amphibia (Ten Eyck, 2008), reptiles (Deckel and Fuqua, 1998), birds (Sperry et al., 2003), and mammals (Olivier and Mos, 1992; Sánchez et al., 1993; Miczek et al., 1998; Simon et al., 1998; De Boer et al., 1999). These data, though consistent, have never attempted to determine whether the serotonergic system is differentially involved in the regulation of the different types of aggression displayed during agonistic interactions.

Weakly electric fish are valuable model systems for the study of agonistic behavior and its neuromodulation. In these animals, behaviors always involve distinct electric displays, in addition to locomotor ones, that depend on a very well-known and tractable electromotor circuit (Stoddard, 2002; Caputi et al., 2005). In different species of electric fish, serotonin is known to modulate both overt aggression and agonistic electric displays (Maler and Ellis, 1987; Capurro et al., 1997; Stoddard et al., 2003; Telgkamp et al., 2007; Allee et al., 2008; Smith and Combs, 2008). We conducted this study to test the role of the serotonergic system in the modulation of agonistic behavior in two syntopic species of South American electric fish that display different types of aggression. *Brachyhypopomus gauderio* (Giora and Malabarba, 2009) is a gregarious weakly electric fish with a polygynous breeding system that exhibits a morphological and electrophysiological sexual dimorphism during the breeding season when intermale aggression is observed (Hopkins et al., 1990; Caputi et al., 1998; Silva et al., 1999; Miranda et al., 2008). *Gymnotus omarorum* (Richer-De-Forges et al., 2009) is a solitary weakly electric fish that displays inter- and intrasexual agonistic behavior across seasons (Black-Cleworth, 1970; Westby, 1975b). The absence of sexual dimorphism and the non-seasonality of its aggressive behavior make this species an advantageous model to evaluate exclusive territorial aggression during the non-breeding season (Batista et al., 2012).

Despite the established role of 5-HT in the modulation of aggression, no studies have explored the effect of 5-HT modulation on the different types of aggression observed within the agonistic context. In this study, we demonstrate differences in the serotonergic modulation of territorial aggression (*G. omarorum*) vs. reproduction-related aggression (*B. gauderio*) based on differential interspecific patterns of serotonergic activity and differential modulation of 5-HT_1A_ receptors.

## Methods

### Subjects and housing

We used sexually-mature male *B. gauderio* (Giora and Malabarba, 2009), formerly *B. pinnicaudatus*, ranging from 16.5 to 22 cm in body length and from 6.5 to 16 g in weight. *B. gauderio* exhibits an obvious morphological sexual dimorphism in tail length and thickness (Hopkins, 1991), that allowed us to easily distinguish males from females. All experiments using *B. gauderio* were performed during the breeding season (December–March). We used non-breeding adults of *G. omarorum* (Richer-De-Forges et al., 2009), formerly *G. carapo*, ranging from 15 to 25 cm in body length and 7.1–50.4 g in weight. Sex in *G. omarorum* is not externally apparent, either morphologically or electrophysiologically, and so was determined after the behavioral experiments by gonadal inspection. All experiments using *G. omarorum* were performed during the non-breeding season (May–August).

*B. gauderio* were obtained from a native population (Laguna Lavalle, 31°48′S, 55°13′W, Department of Tacuarembó, Uruguay) and from a captive-reared colony (Florida International University, Miami, FL, USA). Fish were collected as described elsewhere (Silva et al., 2003) and housed in 500 L outdoor tanks in social groups with the sex ratio observed mid-season in natural breeding colonies (two males and six females, Miranda et al., 2008). *G. omarorum* were collected in Laguna del Sauce (34°51′S, 55°07′W, Department of Maldonado, Uruguay) and housed in individual compartments in 500 L outdoor tanks.

Both species were housed in the outdoor tanks for at least 10 days before the behavioral experiments. Natural photoperiod ranged from around LD14:10 to LD13:11 and from LD10:14 to LD11:13 in breeding and non-breeding seasons, respectively. Water temperature, conductivity, and pH were kept within the normal range they exhibit in the natural habitat (breeding conditions for *B. gauderio* and non-breeding conditions for *G. omarorum*, Silva et al., 2003). Aquatic plants covered the surface of the water and provided shelter for the fish. Fish were fed with *Tubifex tubifex ad libitum*.

All experimental procedures complied with ASAP/ABS *Guidelines for the Use of Animals in Research* and were approved by the Universidad de la República Institutional Ethical Committee (Comisión Bioética, Instituto Clemente Estable, MEC, 007/02/2010), and by the Institutional Animal Care and Use Committee of the Florida International University, Miami, FL (No. 09–023).

### Laboratory settings

Fish were placed in an experimental setup that allowed simultaneous video and electric recordings as described elsewhere (Silva et al., 2007). The experimental tanks, four 50 L glass aquaria (55 × 40 × 25 cm) were fitted with two pairs of orthogonal electrodes attached to each tank wall. The day–night cycle and the physicochemical parameters (water temperature, conductivity, and pH) of the outdoor housing tanks were reproduced in the indoor aquaria. All the experiments were performed during nighttime, and illuminated by an array of infrared LEDs (L-53F3BT, Fablet & Bertoni Electronics) located above the tank (Hupé and Lewis, 2008; Perrone et al., 2009). An infrared-sensitive video camera (SONY CCD-Iris or RoHS CCD) was focused upon the bottom of the tank. The electric signals of freely moving fish were detected by two pairs of fixed electrodes, connected to two high-input impedance amplifiers (FLA-01, Cygnus Technologies Inc.). Images and electric signals were captured by a video card (Pinnacle Systems, PCTV-HD pro stick) or DVD recorded (Philips HDMI), and stored in the computer for further analysis. The fish remained in the recording tank at constant temperature (20–22°C in the non-breeding season; 27–29°C in the breeding season) for 4 h before the beginning of the experiment.

### Behavioral experimental procedures

#### Gymnotus omarorum

***Gate protocol (I).*** We tested the territorial aggression of *G. omarorum* by a gate protocol in which both fish were placed in equally-sized compartments separated by a removable glass gate. We conceived territory as space, as the mass of water fish can navigate around. The gate protocol is the most exigent to test territorial aggression independent of any other motivational drive. This protocol ensures that territory is the only resource that individuals fight for during the non-breeding season and provides symmetric resources and resource values for both contestants: equally-sized plain territory, same residence time, and non-sex-biased dominance, same previous experience (Batista et al., 2012). To be able to predict the contest outcome and to compare the results obtained with and without pharmacological manipulations, we used dyads whose body weight difference ranged from 6 to 30% (*n* = 12). The gate was raised 10 min after sunset, and fish were separated 10 min following conflict resolution. Dominant and subordinate fish were then removed from the tank, killed with an overdose of 0.1% 2-phenoxyethanol, except for those used for the neurochemical analysis which was mechanically killed, and their sex was identified by visual gonadal inspection.

#### Brachyhypopomus gauderio

In *B. gauderio*, social electric displays within reproductive and agonistic contexts show seasonal changes and only occur during the breeding season (Perrone et al., 2009). We focused on breeding inter-male dyadic encounters and evaluated three different experimental protocols aimed to identify the defended resource (territory and/or mate) and to determine the type of aggression displayed by males of this gregarious species: territorial, rank-related, or reproduction-related.

**Figure 1:**
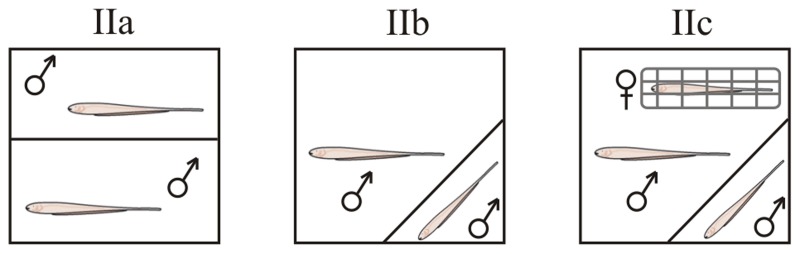
**Behavioral protocols in *B. gauderio*.** Gate protocol (IIa), Resident - Intruder protocol (IIb), Resident - Intruder protocol with female present (IIc).

We used dyads of sexually mature males ranging from 0 to 34% of body weight difference and from 0 to 23% of body length difference. To test the reproductive status of the males, each male was recorded with a female the night before the experiment. Only the males that courted the female during the first night (i.e., those that exhibited 2 of these 3 traits: locomotor courtship displays, nocturnal increase of electric organ discharge (EOD) basal rate, and emission of chirps, Perrone et al., 2009) were tested during the second night using one of the following protocols (Figure 1).

***Gate protocol (IIa).*** Four hours before the test both pre-tested males were placed in equally-sized compartments separated by a removable gate. We evaluated territorial aggression in inter-male dyadic encounters of *B. gauderio* using the same gate protocol used in the *G. omarorum* (protocol I, *n* = 8).

***Resident-intruder protocol (IIb).*** As territory may not be enough to trigger aggression in this gregarious species, we used an asymmetric protocol that enhanced the motivation of the resident toward aggression, by introducing a smaller naïve intruder male to the home tank of a larger pre-tested “resident” male (*n* = 8). In this way, the resident male had greater fighting abilities than the intruder because of his greater size and his enhanced perception of the resource's value having recently courted a female in that same tank. Thirty minutes after sunset the female was removed and the intruder male was added to the tank 5 min later. Fish were separated 10 min following conflict resolution.

***Resident-intruder with female protocol (IIc).*** To test if the presence of the female is necessary to obtain inter-male aggression in this gregarious species, we used a third procedure based on protocol IIb but keeping the female netted in the tank during the experiment (*n* = 8). If protocol IIa elicits aggression, territorial aggression would be the most likely type of aggression displayed by *B. gauderio*. Protocols IIb and IIc are both meant to test reproduction-related aggression, but if only protocol IIc elicited aggression, then the type of aggression *B. gauderio* displays would be a more specific mate-directed competition.

### 8-OH-DPAT administration

The pharmacological manipulation of agonistic behavior was performed using protocol I in *G. omarorum* (*n* = 12) and protocol IIc in *B. gauderio* (*n* = 8). The 5-HT_1A_ agonist 8-hydroxy-di-n-propylamino tetralin (8-OH-DPAT) was purchased in Tocris Cookson Inc. (Ballwin, MO, US). 8-OH-DPAT is a specific agonist of the 5-HT_1A_ receptor in mammals (Middlemiss and Fozard, 1983), and other vertebrates (Barrett, 1992; Khan et al., 1996; Winberg and Nilsson, 1996). One hour before the encounter, expected dominants (resident larger male in *B. gauderio* and larger individual in G. *omarorum*) were injected either intraperitoneally or intramuscularly with 1 μl/g body weight of a 2.5 mM 8-OH-DPAT solution dissolved in physiological saline as previously described (Stoddard et al., 2003; Allee et al., 2008).

### Behavioral data processing

#### Locomotor displays

Video recordings of agonistic encounters were analyzed following Batista et al. (2012), using the same criteria in both species. We identified the three phases of agonistic encounters in both species: (1) pre-contest phase: from time 0 (gate removal in gate protocols and intruder addition in resident-intruder protocols) to the occurrence of the first attack; (2) contest phase: from the occurrence of the first attack to conflict resolution (resolution time); and (3) post-resolution phase: 10 min after conflict resolution. Conflict resolution was defined as the moment we observed the third consecutive retreat of one fish without retaliating. This criterion unambiguously defined subordination status; fish fulfilling this requirement were never observed to change their status in the following 10 min of interaction. We calculated attack rate dividing the number of attacks [bites, nips, nudgings (Black-Cleworth, 1970)] by contest duration time in seconds.

#### Electric signals

We measured the occurrence and timing of signal cessations or “offs,” and transient increases in EOD rate with waveform distortion or “chirps” in *B. gauderio* control agonistic encounters, following Batista et al. (2012). We analyzed the effects of 8-OH-DPAT injection (to the expected dominant) upon offs and chirps in *B. gauderio* but not in *G. omarorum*, in which these electric displays are only emitted by subordinates. The first off latency and first chirp latencies were calculated as the times to first off or chirp minus the time of occurrence of the first attack.

### Analysis of monoamines

We quantified 5-HT and 5-HIAA in dominants and subordinates of both *G. omarorum* (*n* = 7 dyads) and *B. gauderio* (after protocol IIc, *n* = 7 dyads). As a control group, we used isolated individuals of both species subjected to similar experimental manipulation without the opportunity to fight (*G. omarorum, n* = 7; *B. gauderio, n* = 6). Ten minutes after conflict resolution, fish were separated, kept isolated for 1 h, and then rapidly netted, decapitated, and their brains were dissected. Brain samples were wrapped in aluminum foil, frozen, and kept at −80°C. 5-HT and 5-HIAA levels in telencephalon (excluding the olfactory bulb) were quantified using HPLC with electrochemical detection equipped with a C-18 column (5 μm particles, 150 × 4.6 mm, Phenomenex, USA) and an electrochemical detector (LC-4C BAS). 5-HT and 5-HIAA were calculated as ng per g wet tissue. The ratio of 5-HIAA/5-HT was calculated indicating the serotonin turnover activity on telencephalic tissue.

### Statistics

All locomotor, electrical, and tissue data were analyzed with non-parametric tests: Wilcoxon Matched-Pairs test (paired variables in the same fish or both fish in a dyad), Mann–Whitney U test (independent variables using sets of data from different fish), and Kruskal–Wallis test (independent variables using three sets of data from different fish). For this reason, data are expressed as median and median absolute deviation (MAD) throughout. We used Chi-square tests (χ^2^) to test effect of 8-OH-DPAT administration upon contest outcome.

## Results

### Agonistic behavior in *Brachyhypopomus gauderio*

Under the gate protocol behavioral test (protocol IIa), meant to evaluate if acquisition of additional territory is sufficient incentive to elicit aggression, only 2 of 8 inter-male dyads engaged in agonistic behavior and only one of the conflicts was resolved. Though these males exhibited courtship behavior the night before, when exposed to another male they showed exploratory behavior and little direct interaction. In contrast, inter-male dyads created under the resident-intruder protocols (protocol IIb, IIc) engaged in fights in all 16 trials, regardless of whether a female was present (protocol IIc) or absent (protocol IIb). We always observed conflict resolution and the resident male won the fight in 15 of 16 cases. Moreover, we found no differences between protocols IIb and IIc in several parameters of agonistic encounters: first attack latency, contest duration, resident attack rate, and intruder attack rate (Table 1).

**Table 1:** **Comparison between Resident–Intruder protocols in *B. gauderio*: Resident–Intruder protocol (IIb), Resident–Intruder protocol with female present (IIc)**.

	**Protocol IIb**		**Protocol IIc**		
	**Median**	**MAD**	**Median**	**MAD**	***p***
1st Attack latency (s)	458.5	395.5	309	184	0.96
Contest duration (s)	681.5	329.5	379	209.5	0.19
Resident attack rate	0.025	0.0059	0.03	0.01898	0.37
Intruder attack rate	0.0033	0.003	9E-5	8.6E-4	0.96

Mann–Whitney U test. No significant differences were found at p < 0.05.

**Figure 2:**
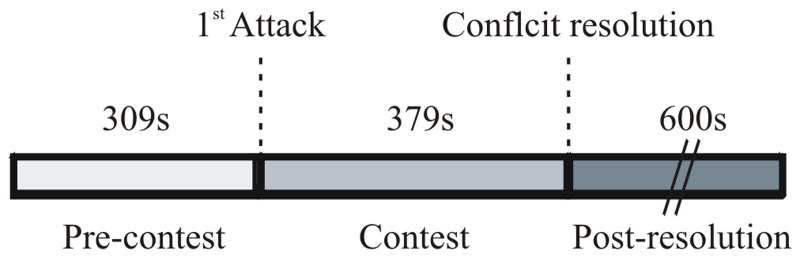
**Dynamics of agonistic encounters in *B. gauderio* using protocol IIc, The size of the bars represents median values (*n* = 8)**.

As shown in Figure 2, agonistic encounters in *B. gauderio* followed the three standard phases: pre-contest (first attack latency = 512.5 ± 497 s), contest (contest duration = 421.5 ± 527 s), and post resolution. Within the contest phase, we observed species-specific locomotor displays of overt aggressive behaviors; for example, mutual attempts to bite the contender's tail in antiparallel position. Social electric signals, chirps (in 3 of 8 dyads) and offs (in 1 of 8 dyads), were observed during the contest and post-resolution phases but these electric displays did not seem to follow a predictable temporal pattern. We recorded several types of chirps that were either produced by dominants (types A and M, Perrone et al., 2009) or subordinates (type B, Perrone et al., 2009). We only recorded few offs that were always produced by the subordinate fish.

### Interspecific differences in the action of 5-HT_1A_ agonist on aggression

The 5-HT_1A_ receptor agonist 8-OH-DPAT significantly affected the territorial aggression of *G. omarorum*, but did not affect *B. gauderio* inter-male aggression tested by protocol IIc (Table 2). The administration of 8-OH-DPAT to the expected winner in *G. omarorum* caused a shift in the contest outcome. After the administration of the 5-HT agonist, the dominant could no longer be predicted by size asymmetry as previously demonstrated by Batista et al. (2012), (χ^2^ test, *p* = 0.027). The administration of 8-OH-DPAT also decreased the attack rate of the injected fish compared to the attack rate shown by the larger fish in control dyads (Mann–Whitney U test, *p* = 0.03). However, 8-OH-DPAT had no discernable effect on the 1st attack latency (Mann–Whitney U test, *p* = 0.52) nor contest duration (Mann–Whitney U test, *p* = 0.4) in this species. In *B. gauderio*, injection of 8-OH-DPAT to the resident male did not affect contest outcome (χ^2^ test, *p* = 1), attack rate of the injected fish (Mann–Whitney U test, *p* = 0.75), 1st attack latency (Mann–Whitney U test, *p* = 0.6), nor contest duration (Mann–Whitney U test, *p* = 0.4).

**Table 2:** **Effects of 8-OH-DPAT upon outcome, attack rate of the injected fish, 1st attack latency, and conflict duration in *B. gauderio* (*n* = 8) and *G. omarorum* (*n* = 12)**.

	***B. gauderio***	***p***	***G. omarorum***	***p***
**OUTCOME**				
Control	Resident 8/8	1	Large 11/12	0.027^*^
8-OH-DPAT	Resident 7/8		Large 5/12		
**INJECTED FISH ATTACK RATE**				
Control	0.03 (0.019)	0.75	0.12 (0.055)	0.03^*^
8-OH-DPAT	0.027 (0.014)		0.045 (0.026)		
**1ST ATTACK LATENCY (S)**				
Control	309 (184)	0.6	29 (18.5)	0.52
8-OH-DPAT	634 (439.5)		45 (23.5)		
**CONTEST DURATION (S)**				
Control	379 (209.5)	0.4	136 (71.5)	0.4
8-OH-DPAT	426 (311)		109 (70)		

B. gauderio protocol IIc. Values expressed as Median (MAD).

* p < 0.05; p values refer to the comparison between control and 8-OH-DPAT in B. gauderio and G. omarorum respectively. Outcome was tested by Chi^2^test; the other variables were tested by Mann–Whitney U test.

### Interspecific differences in serotonergic activity

**Figure 3:**
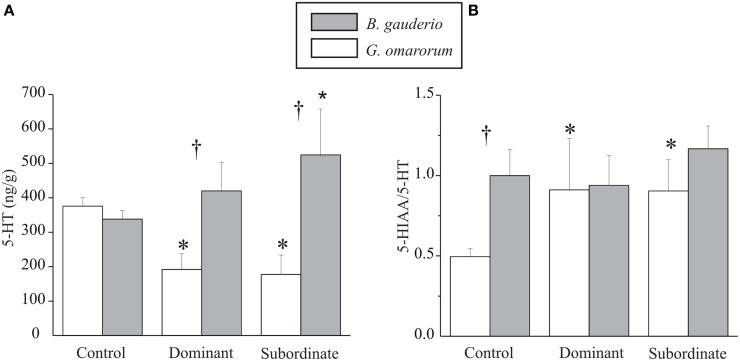
**Telencephalic levels of 5-HT and serotonergic activity after conflict resolution. (A)** Levels of 5-HT (ng/g) and **(B)** 5-HT turnover (5-HIAA/5-HT ratio) 1 h after conflict resolution in controls, dominants, and subordinates of *B. gauderio* (protocol IIc) and *G. omarorum* (protocol I). Kruskal–Wallis test analyzed intraspecific comparison; ^*^p < 0.05 when tested against controls. Mann–Whitney U test analyzed interspecific comparison; ^†^*p* < 0.05.

As shown in Figure 3A, subordinate males of *B. gauderio* exhibited significantly higher telencephalic 5-HT levels than control males (Kruskal–Wallis test, *p* = 0.027) whereas no significant differences were found either between dominants and controls (Kruskal–Wallis test, *p* = 0.43) or between dominants and subordinates (Kruskal–Wallis test, *p* = 0.25). In this species, serotonergic turnover measured by 5-HIAA/5-HT ratio, showed no significant differences between subordinates, dominants, and controls (Kruskal–Wallis test, *p*_dominants-controls_ = 0.52, *p*_subordinates-controls_ = 0.83, *p*_dominants-subordinates_ = 0.37, Figure 3B). In contrast, territorial aggression in *G. omarorum* exhibited the completely opposite pattern of 5-HT levels and serotonin turnover after conflict resolution (Figure 3): 5-HT levels in both subordinates and dominants were significantly lower than controls (Kruskal–Wallis test, *p*_dominants-controls_ = 0.0015 and *p*_subordinates-controls_ = 0.002) but not significantly different between them (Kruskal–Wallis test, *p*_dominants-subordinates_ = 0.56). Serotonin turnover was different between dominants and subordinates with respect to controls (Kruskal–Wallis test, *p*_dominants-controls_ = 0.056 and *p*_subordinates-controls_ = 0.009), but no difference was found between dominants and subordinates 5-HIAA/5-HT ratio (Kruskal–Wallis test, *p* = 0.87). If we now focus on the interspecific comparison, both dominants and subordinates of *B. gauderio* exhibited higher levels of 5-HT after fights than *G. omarorum* (Mann–Whitney U test, *p*_dominants_ = 0.024, and *p*_subordinates_ = 0.0014, Figure 3A), although basal levels of 5-HT did not differ between species (Mann–Whitney U test, *p* = 0.43, Figure 3A). Basal 5-HIAA/5-HT ratio was higher in *B. gauderio* than in *G. omarorum* (Mann–Whitney U test, *p* = 0.003, Figure 3B), whereas we found no differences between subordinates and dominants of both species (Mann–Whitney U test, *p*_dominants_ = 0.69, *p*_subordinates_ = 0.18, Figure 3B).

## Discussion

This is the first study that presents evidence on the differential participation of the serotonergic system in the modulation of two types of aggression: the non-breeding territorial aggression of *G. omarorum* and the reproduction-related aggression of males *B. gauderio*. This finding was only possible because each study species displayed a distinctive type of aggression as part of its natural behavioral repertoire, which we were able to identify unambiguously using a very careful experimental design.

### Weakly electric fish as model systems of different types of aggression

Both pioneer and recent studies have described territorial aggression in the genus *Gymnotus* (Black-Cleworth, 1970; Westby, 1975a; Batista et al., 2012). As in other members of this genus, an empty space can hold value and is defended vigorously in *G. omarorum*. This species exhibits a very clear-cut example of territorial aggression, in which in dyadic encounters during the non-breeding season, territory is not only enough to elicit aggression, it is the only defended resource (Batista et al., 2012). This example of non-sex biased aggression, fairly unique among teleosts, is probably the bases for the establishment of individual territories in natural populations of *G. omarorum*.

When we tested competence over a plain territory in *B. gauderio* (protocol IIa) we confirmed that breeding inter-male aggression in *B. gauderio* is not exclusively territorial, as the possession of equal resources was not enough to elicit aggression. We only observed aggression when the resource value was experimentally increased by residency (protocols IIb and IIc). We postulate that the most likely type of aggression displayed by *B. gauderio* is reproduction-related aggression. *B. gauderio* is a gregarious species with a polygynous breeding system in which males hold consistent home ranges typical of exploded lek polygyny (Miranda et al., 2008). It is likely that the site each male occupies is established by competition since the males possessing optimal sites within the lek have more chances to breed (Schuster and Wade, 2003). As in other gymnotiforms (Hopkins, 1974; Hagedorn and Heiligenberg, 1985; Zakon et al., 1991; Fugère et al., 2011), *B. gauderio* establishes ranks of EOD rate, in which the dominant fish increases its discharge rate after contest resolution (unpublished data). In this way, a fish by signaling its dominance avoids time-consuming contests and bruising from fights. Typically, when winning a contest is a proxy of male quality in itself (i.e., a trait to be selected by a female in the audience), mate competition will be increased by the presence of the female and decreased by its absence (Wong and Candolin, 2005). Agonistic encounters with (protocol IIc) or without a female (protocol IIb) in the vicinity were indistinguishable (Table 1). Further, as the presence of the female at the moment of the contest was not required to trigger aggression and did not elicit higher motivation toward aggression nor higher aggression levels, direct competition among males for good habitat (rather than mate-directed competition) is the most likely the type of aggression displayed by *B. gauderio*.

Electric signaling is an advantageous channel to broadcast fighting ability and dominance status modulating rate and amplitude of species-specific electric discharges. It is striking that the differences in agonistic electric communication in gymnotiforms lie not in the diversity of signals but in the different meaning the signal conveys in each species (Black-Cleworth, 1970; Westby, 1975a,b; Hagedorn and Zelick, 1989; Hupé et al., 2008; Triefenbach and Zakon, 2008; Perrone et al., 2009; Fugère and Krahe, 2010; Batista et al., 2012). Further, the role of communicative electric displays should be coherent with the type of aggression displayed and the social structure of each species. For a minimally aggressive gregarious species like *B. gauderio*, it may be important to advertise dominance by electric signaling whereas subordinates may not be forced to signal submission. On the other hand, electrical submission is likely to be expected in *G. omarorum* as a highly aggressive solitary territorial species. Whether it is important in this species to advertise dominance by electric cues remains unclear. To signal dominance in *G. omarorum* may be unnecessary given the seldom intraspecifc interactions. However, as individuals of this species are active territory guards, it may be useful for them to signal dominance at distance.

Though diverse in displays and triggering stimuli, agonistic behavior can be compared among species because it progresses through the same stages of neuroendocrine control and behavioral states [evaluation, contest, and post resolution, (Summers and Winberg, 2006)]. Agonistic encounters in *G. omarorum* (Batista et al., 2012) and *B. gauderio* followed these 3 phases but with different temporal profiles. Both the evaluation and the contest phases were shorter in *G. omarorum* (Batista et al., 2012) than in *B. gauderio* (Figure 2). Moreover, the agonistic encounters differed in ferocity between species i.e., *G. omarorum* used more harmful displays (bites, jaw-locks) and displayed higher levels of aggression (Batista et al., 2012) than *B. gauderio.* These results denote higher aggressive motivation and predisposition and lower tolerance of conspecifics in territorial aggression than in reproduction-related aggression. Solitary animals are less prone to mitigate conflicts, prevent aggressive escalation, or promote reconciliation than gregarious animals that need to live in stable social organizations (Aureli and De Waal, 2000; De Waal, 2000). It was therefore predictable that the gregarious *B. gauderio* engaged in milder contests than *G. omarorum*, and that once the conflicts were resolved, dominants were more tolerant to subordinates.

Water temperature is a major environmental factor influencing the biology of subtropical gymnotiform species (Silva et al., 1999, 2002, 2003; Ardanaz et al., 2001; Quintana et al., 2004). For example, seasonal variation of water temperature is enough to induce gonadal maturation in *B. gauderio* (Quintana et al., 2004). Taking into account the environmental conditions in which it is possible to trigger each type of aggression distinctively, we performed the experiments at different temperatures among species (20–22°C for the non-breeding territorial aggression of *G. omarorum* and 27–29°C for the reproduction-related aggression of *B. gauderio*). In ectothermic animals, temperature deeply influences all the metabolic processes and therefore may affect aggression as well. However, overall temperature effects are not easy to interpret. Higher temperatures tend to increase animals' activity but also trap animals in metabolic threats as the oxygen content of water decreases as temperature increases. Therefore, we can expect either and increase or decrease in aggression as temperatures increases. Though *G. omarorum* exhibits agonisitic behavior all year round, the level of aggression it displays is not significantly different when recorded at around 20°C (non-breeding) or at 28°C (breeding; G. Batista, personal communication).

### Role of 5-HT in the control of different types of aggression

Neither general nor simple causal relationships can be established between serotonergic activity and aggression. From an evolutionary perspective, we now understand that the effects of 5-HT on aggressive behavior are brain region, time, and context-dependent (Summers et al., 2005b). Our understanding of the serotonergic control of aggression requires the analysis of local changes of 5-HT activity and temporal profile in specific brain regions, and careful behavioral procedures to limit our observations to natural species-specific displays. In this study that relies on a thoroughly description of two distinctive types of aggression, we attempted to evidence the differential role of the 5-HT system in the regulation of aggression using a primary approach that combined gross neurochemical data with pharmacological manipulation of 5-HT_1A_ receptors. In short, the territorial aggression of *G. omarorum* is characterized by a low basal serotonergic activity and a slow recovery of 5-HT levels after fights, which is consistent with the clear anti-aggressive effect of the 5-HT_1A_ agonist obtained with the pharmachological manipulation in this species. On the other hand, the reproduction-related aggression of *B. gauderio* shows a dynamic response of the serotonergic system, in which subordinates exhibit the expected higher 5-HT levels than controls. The serotonergic system in *B. gauderio* is already highly active in basal conditions, which may explain the non-responsiveness of reproduction-related aggression after 5-HT_1A_ agonist administration.

#### Telencephalic serotonergic activity

After inter-male encounters of *B. gauderio*, subordinate males (but not dominants) exhibited significantly higher telencephalic levels of 5-HT (Figure 3A) and of 5-HIAA (data not shown) than controls. The 5-HIAA/5-HT ratio did not show a significant difference between dominance classes (Figure 3B) nevertheless, the fact that both 5-HT (Figure 3A) and 5-HIAA (data not shown) levels increased allow us to conclude that serotonergic activity increases in subordinate males following a contest. Taken together, we interpret these data as the expected pattern of serotonergic activation suppressing aggression in subordinate individuals (Summers et al., 2003, 2005b; Summers and Winberg, 2006). Interestingly, the serotonergic response to an aggressive interaction is different in the territorial aggression displayed by *G. omarorum* than in the reproduction-related aggression of *B. gauderio*. In first place, the expected involvement of the serotonergic system in the control of aggression in *G. omarorum* was confirmed by the significant increase in the 5-HIAA/5-HT ratio observed in subordinates' telencephalon (Figure 3B). The fact that dominants also increased their 5-HT turnover (Figure 3B) cannot be interpreted as an unusual trait because an early increase in dominants 5-HT turnover has been reported elsewhere (Overli et al., 1999; Summers et al., 2003). 5-HT levels exhibited by both subordinates and dominants of *G. omarorum* after territorial aggression were significantly lower than controls (Figure 3A), possibly caused by an increased release and delayed synthesis of 5-HT in *G. omarorum*.

With the exception of a comparative study among two species of macaques (Westergaard et al., 1999), no previous reports compare the activity of the serotonergic system between species with different social structure or displaying different types of aggression. There have been more attempts to associate the differential involvement of 5-HT in different behavioral phenotypes within a same species. In the lizard *Anolis carolinensis*, males that show more aggressive potential exhibit lower serotonergic activity in specific brain regions than non-aggressive males (Summers et al., 2005b). Serotonergic activity is also different between the excessively aggressive short attack latency (SAL) and the long attack latency (LAL) mouse lines; after repeated resident-intruder fighting experience, 5-HT levels in the prefrontal cortex of SAL mice are significantly lower than in LAL mice (Caramaschi et al., 2008; De Boer et al., 2009).

We found interspecific differences in the serotonergic system that were not reflected in the basal 5-HT levels but on the dynamic profile of the changes in 5-HT levels from pre-contest to post-contest. Basal levels of 5-HT, quantified in animals that did not engage in aggressive interactions (controls), are similar between species (Figure 3A), allowing us to assume that interspecific differences do not reside in 5-HT availability. However, the fact that the 5-HIAA/5-HT ratio is lower in *G. omarorum* than in *B. gauderio* leads us to conclude that the basal activity of the serotonergic system is different between species (Figure 3B). This result is in accordance to the 5-HT deficiency hypothesis that, if applied to this interspecific framework, would predict that the more aggressive species is expected to have less serotonergic basal activity. Moreover, when the aggressive interaction takes place, we observed that dominants and subordinates show lower levels of 5-HT after the territorial aggression displayed by *G. omarorum* than after the reproduction- related aggression of *B. gauderio* (Figure 3A).

#### Pharmacological modulation of aggression by 5-HT_1A_ receptor agonist

The involvement of 5-HT_1A_ receptors in the serotonergic inhibition of aggression is indisputable across vertebrates (Nelson and Trainor, 2007). As expected, the administration of 8-OH-DPAT to the putative winner in *G. omarorum* decreased aggression in the injected fish (Table 2). Moreover, this maneuver caused a general distortion of the normal agonistic encounter that even reversed the expected outcome, a phenomenon that has been associated to 5-HT changes in other vertebrates (Summers et al., 2005a). The inhibition of aggression previously reported in this species by pharmacological manipulation of 5-HT levels (Capurro et al., 1997) is now confirmed to be mediated (at least partially) by 5-HT_1A_ receptors. On the other hand, the administration of 8-OH-DPAT to the expected winner (resident male) in *B. gauderio* did not affect any aspect of the agonistic encounter. Previous reports in *B. gauderio* showed that 8-OH-DPAT diminishes the amplitude and duration of the EOD waveform (Allee et al., 2008), which is an important signal of male body size and endocrine status (Gavassa et al., 2011, 2012). The unexpected lack of effect of the 5-HT_1A_ agonist in *B. gauderio* is nevertheless relevant because: (1) it is the first report to our knowledge in which the administration of 5-HT_1A_ receptor agonists do not affect aggression in realistic dyadic encounters; and (2) it strengthens the body of data that show interspecific differences in the organization of the 5-HT system. Even if the presence of 5-HT_1A_ receptors has been documented in fish, amphibians, reptiles, and birds (Palacios and Dietl, 1988; Gleeson et al., 1992; Lima et al., 1992; Khan et al., 1996; Winberg and Nilsson, 1996; Marracci et al., 1997; Norton et al., 2008), a comparative analysis of the degree of conservation of the receptor across taxa has not been performed. However, in both, *Danio rerio* and *Xenopus laevis*, the 5-HT_1A_ receptors show an overall amino-acid identity of 78 and 73% respectively compared to the mammal receptor (Marracci et al., 1997; Norton et al., 2008). We thus assume that 8-OH-DPAT is acting specifically on the 5-HT_1A_ receptors in *G. omarorum* and *B. gauderio*, but further experiments will be necessary to validate this assumption.

The interspecific differences we found in the modulation of aggression by the 5-HT_1A_ receptor agonist may be attributed to: (a) the impossibility of 8-OH-DPAT to decrease an already low attack rate displayed by *B. gauderio*; (b) differences in pre and post-synaptic 5-HT_1A_ receptor density; (c) differential 5-HT_1A_ receptor sensitivity among species; and (d) relative balance between interspecific differences in the serotonergic basal activity and the fixed dose of 8-OH-DPAT we used. For example, the excessively aggressive mice line SAL show higher sensitivity of the 5-HT_1A_ autoreceptors than the more docile LAL line, and 8-OH-DPAT likewise had more effect on SAL mice (Caramaschi et al., 2007).

### Speculations and perspectives

Serotonergic control of agonistic behavior differs between the solitary *G. omarorum* and the gregarious *B. gauderio*. As each species displays a different type of aggression within its natural agonistic context (territorial vs. reproduction-related aggression, respectively), the species-specific organization of the serotonergic system may be related to each aggression type. Moreover, the differences we found in the serotonergic system match coherently with the social structure of each species, and may be interpreted as being positively selected according to each ecological context. *G. omarorum*, a solitary and highly aggressive predator, is a species meant to always be alert. Hence, in natural occurring contests, when the loser flees from a defended site, it may not be necessary and might even be maladaptive to have continued aggression inhibited by high 5-HT levels. On the other hand, a male *B. gauderio* who loses a boundary dispute may remain close to the winning male after conflict resolution; to inhibit aggression and new aggressive challenges by keeping high 5-HT levels may therefore constitute an evolutionary stable strategy in this species.

We currently understand that the wide diversity in social behavior observed among vertebrates resides on the plasticity of the weighting of activity across evolutionary conserved neural networks (Newman, 1999; Goodson and Kabelik, 2009; O'Connell and Hofmann, 2011, 2012). Within this framework, we may speculate whether the differences we found in the organization of the serotonergic system regarding different types of aggression reveal a more general strategy observable in other vertebrates. For example, is there a specific pattern of activity of the serotonergic system associated with each type of aggression across vertebrates? Has the evolution of different neural strategies in the organization of the serotonergic system shaped the association between different sociality and types of aggression? Although this study cannot discern these questions, it is a good starting point to promote further studies to test these hypotheses in the future.

### Conflict of interest statement

The authors declare that the research was conducted in the absence of any commercial or financial relationships that could be construed as a potential conflict of interest.
